# THE BENEFITS OF GENERATION IDENTITY OVER AGE IDENTITY IN OLDER ADULTS: THE MEDIATING ROLE OF NOSTALGIA

**DOI:** 10.1093/geroni/igae098.3737

**Published:** 2024-12-31

**Authors:** Yuanqing Chang, Jiehua Lu, Xin Zhang

**Affiliations:** Peking university, Peking University, Beijing, China (People’s Republic); Peking university, Peking University, Beijing, China (People’s Republic); Peking University, Beijing, Beijing, China (People’s Republic)

## Abstract

**Background and Objectives:**

As individuals age, they develop two types of self-identification: age identity and generation identity. Previous research suggests generation identity is more beneficial in promoting well-being of older adults. However, the underlying mechanisms and the generalizability in the Chinese cultural context remain underexplored.

**Research Design and Methods:**

This study employed a randomized single-factor between-subjects design with 160 community-dwelling Chinese older adults (M = 67.61 years, SD = 5.63 years; 31.88% men). Participants were randomly assigned to focus on similarities or differences with peers (age identity salience condition) or contemporaries (generation identity salience condition), followed by assessments of nostalgia and subjective well-being including life satisfaction, loneliness, meaning in life, and awareness of age-related changes.

**Results:**

Word frequency based on lexicon text analysis revealed that participants in generation identity salience condition mentioned more era-specific events. The Biterm Topic Model (BTM) identified two themes: “Hardship of the Past vs. Present Happiness” and “Present Happy Life” in both groups. However, the generation group had a higher proportion of the first theme (56.25% vs. 42.5%). Independent T-tests indicated significant group differences in nostalgia and subjective well-being (p <.05). Structural equation modeling revealed nostalgia could function as a significant mediator between identity salience condition and subjective well-being (β =.092, 95% CI = [0.000, 0.184]).

**Discussion and Implications:**

The findings suggest that emphasizing generation identity can significantly enhance older individuals’ life satisfaction, meaning in life, and awareness of age-related gains, offering empirical support for strategies aimed at promoting active and successful aging.

